# Making the case for better integration of cervical cancer screening and treatment for HIV-infected women attending care and treatment clinics in Dar es Salaam, Tanzania

**DOI:** 10.1186/1750-9378-7-S1-P13

**Published:** 2012-04-19

**Authors:** Renicha McCree-Hale, Donna Spiegelman, Eric Aris, Guerino Chalamilla, Irene Andrew, Julius Mwaiselage, Lisa Hirschhorn, Wafaie Fawzi

**Affiliations:** 1 Department of Health Behavior, University of Alabama at Birmingham, Birmingham, AL, USA; 2 Departments of Epidemiology and Biostatistics, Harvard University, Boston, MA, USA; 3 Management and Development for Health, Harvard PEPFAR Program, Dar es Salaam, Tanzania; 4 Ocean Road Cancer Institute, Dar es Salaam, Tanzania; 5 Department of Medicine, Harvard Medical School, Boston, MA, USA; 6 Departments of Epidemiology, Nutrition, and Global Health, Harvard University, Boston, MA, USA

## Background

HIV infected women are more likely to have persistent oncogenic human papillomavirus infections that lead to precancerous cervical lesions and cervical cancer. Of the incident cases of cervical cancer in Africa, 40% occur in East Africa [5]. Tanzanian women bear the highest burden of cervical cancer in the region with age adjusted standardized incidence and mortality rates of 50.9 and 37.5 cases per 100,000 women. Cervical cytology was performed to estimate the prevalence of squamous intraepithelial lesions (SIL) and determine patient follow up for treatment of histologically confirmed SIL.

## Methods

Between December 2006 and August 2009, physicians in HIV care and treatment clinics in Dar es Salaam, Tanzania performed conventional PAP smears on 1440 women who voluntarily accepted a cervical screening. Slides were prepared and sent to the histopathology lab at the Muhimbili National Hospital, Dar es Salaam, for examination. Positive smears included the detection of low-grade SIL (LSIL) and high-grade SIL (HSIL). Negative smears were defined by the detection of atypical squamous cells of undetermined significance and normal results.

## Results

A total of 1440 smears were examined, and 124 (8.61%) of women had SIL. On cytology, 72 (5%) had LSIL and 49 (3.4%) had HSIL. On histology, 889 (61.74%) of all women screened had cervicitis or inflammation. Of those who were positive for SIL, 95 (76.61%) had cervicitis or inflammation. None of the women were found to have invasive cancer. Of the 124 women with SIL, 5 (4%) presented for follow up and treatment at the national cancer center in Dar es Salaam. The remaining 119 women had to be tracked using a district tracking mechanism comprised of trained lay health workers.

## Conclusion

The findings indicate a need for better integration of cervical cancer prevention for women attending HIV care and treatment clinics. Even in highly efficient HIV clinics with good health services, cervical screening programs based on cytology do not provide adequate screening coverage and timely access to treatment. *Single visit* models including immediate treatment with cryotherapy are more effective and context appropriate. However, innovative patient retention approaches will likely be necessary for treatment procedures that do not meet the criteria for cryotherapy and require follow up at the national cancer center. Implementation research will be needed to identify novel and sustainable approaches for comprehensive service delivery of cervical cancer screening and treatment in the context of HIV care and treatment clinics.

